# Nonlinear Deblurring for Low-Light Saturated Image

**DOI:** 10.3390/s23083784

**Published:** 2023-04-07

**Authors:** Shuning Cao, Yi Chang, Shengqi Xu, Houzhang Fang, Luxin Yan

**Affiliations:** 1 The National Key Laboratory of Science and Technology on Multispectral Information Processing, School of Artificial Intelligence and Automation, Huazhong University of Science and Technology, Wuhan 430074, China; sn_cao@hust.edu.cn (S.C.);; 2 The Artificial Intelligence Center, Peng Cheng Laboratory, Shenzhen 518055, China; 3 The School of Computer Science and Technology, Xidian University, Xi’an 710071, China

**Keywords:** nonlinear model, low-light saturated images, ringing artifact, image deblurring

## Abstract

Single image deblurring has achieved significant progress for natural daytime images. Saturation is a common phenomenon in blurry images, due to the low light conditions and long exposure times. However, conventional linear deblurring methods usually deal with natural blurry images well but result in severe ringing artifacts when recovering low-light saturated blurry images. To solve this problem, we formulate the saturation deblurring problem as a nonlinear model, in which all the saturated and unsaturated pixels are modeled adaptively. Specifically, we additionally introduce a nonlinear function to the convolution operator to accommodate the procedure of the saturation in the presence of the blurring. The proposed method has two advantages over previous methods. On the one hand, the proposed method achieves the same high quality of restoring the natural image as seen in conventional deblurring methods, while also reducing the estimation errors in saturated areas and suppressing ringing artifacts. On the other hand, compared with the recent saturated-based deblurring methods, the proposed method captures the formation of unsaturated and saturated degradations straightforwardly rather than with cumbersome and error-prone detection steps. Note that, this nonlinear degradation model can be naturally formulated into a maximum-a posterioriframework, and can be efficiently decoupled into several solvable sub-problems via the alternating direction method of multipliers (ADMM). Experimental results on both synthetic and real-world images demonstrate that the proposed deblurring algorithm outperforms the state-of-the-art low-light saturation-based deblurring methods.

## 1. Introduction

**Figure 1:**
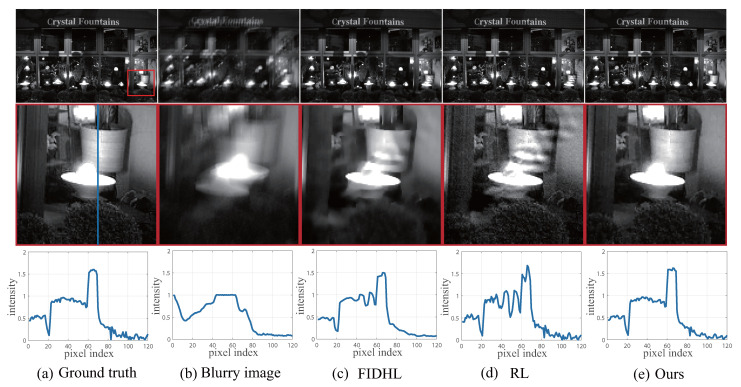
Illustration of the ringing artifacts in saturation deblurring. The first row is the restored images. The second row is the close-ups of the restored images. The third row is the cross profile of columns through close-up along the blue line. The first and second columns are (**a**) the ground truth and (**b**) blurry image. The third to last columns are the deblurring results of the linear deblurring method by (**c**) FIDHL [25], (**d**) RL [36], and (**e**) the proposed nonlinear method. The ringing artifacts **near the saturation region** are widely observed in the linear methods.

Blurry images with saturation are common in our daily life, especially when taken with hand-held equipment, including smartphones in low-light conditions as shown in Figure 1a. Saturation happens when the radiance of the captured scene exceeds the limited range of the camera sensor, leaving the pixel intensity clipped at the maximum output value, such as 255 for an 8-bit image. Most existing deblurring methods [1,2,3,4,5,6,7,8,9] assume the linear degradation model:

(1) y=Kx+n,

where $y\in R^{N}$, $x\in R^{N}$, and $n\in R^{N}$ are a blurry image, latent image, and noise, respectively, and $K\in R^{N\times N}$ is the blurring matrix corresponding to the convolution of blur kernel k.

The linear model (1) is custom-designed for formulating the blur degradation of natural image. However, it is not effective in handling blurry images with significant saturation, since it usually leads to severe ringing artifacts in the deblurring results. The reason for the low-quality restoration is that the saturated pixels violate the linear degradation model (1), and errors are introduced in the process of deblurring. The saturation results in ringing artifacts during the deconvolution procedure, and the real intensity of saturated pixels is dramatically higher than for adjacent pixels.

The recent saturation deblurring methods [10,11,12,13,14,15,16,17,18,19,20,21] tend to detect and discard the saturation regions, and the true intensity of the saturation regions is estimated with the prior assumption of the latent clean image; the commonly used image priors include TV and $l_{p}$ norm gradient prior [12,14]. Unfortunately, these methods are sensitive to the detection accuracy of the saturation and prone to lose useful information in the transition region. Instead of the detection–restoration paradigm, certain methods [12,13,14,16,17] also assign saturation with small weights according to the iteration error to constrain the negative effect of the saturation.

Another line of work [15,22] argued that high-light streaks caused by point-light sources, which are different from large area saturation light sources, can provide rich motion information for motion blur kernel estimation. This is because the light streaks have the same shape as the underlying blur kernels. However, this assumption may not always hold true, especially when there are no ideal light streaks in the real scene.

In this paper, to address the general saturation case—in particular, for a large saturated area—we propose a unified framework that models the saturated and natural areas simultaneously. To do this, we first, analyze the failure, namely the ringing artifacts of the conventional linear based deblurring methods (Section 3.1.1). We observe that the ringing artifacts are always due to the estimation errors in the saturation region propagated to the natural region (Section 3.1.2).

This motivates us to divide an image into three areas, including saturated, transition (the unsaturated pixels adjoin to the saturated regions, whose intensity is enhanced by the saturated pixels due to the blending of the blur process), and natural areas (the unsaturated pixels are far away from the saturated regions, whose intensity gains no influence from the saturated pixels). Thus, we design a nonlinear piecewise function to model the saturation, natural pixels, and also the transition pixels between them simultaneously (Section 3.1.3). For the natural region, we keep them unchanged as in the linear model; for the saturation region, we truncate the max intensity as 1; and for the transition region, we introduce a monotonic increasing function to fit the gradual intensity change between natural and saturation pixels (Section 3.2.1).

Compared with the previous low-light saturated images deblurring methods, the proposed nonlinear deblurring method does not need any cumbersome and error-prone detection steps [10,11] or heuristic operations, such as the iteration error re-weight [12,13,14,16,17]. On the contrary, by enforcing the nonlinear function on the conventional linear model (1), our framework offers a unified perspective with clear physical meanings for both the unsaturation and saturation blurry image. Based on the nonlinear degradation model, we propose the nonlinear deblurring method, which can be well modeled within the maximum-a-posterior (MAP) framework (Section 3.2.3).

The contributions of this paper can be summarized as follows.

- We analyze the ringing artifacts caused by estimation errors in the saturation region and introduce a nonlinear function to separate errors in different regions, thereby, reducing the ringing artifacts caused by error-prone estimated saturation regions.
- We propose a unified nonlinear deblurring method within the MAP framework, which can be efficiently solved.
- The proposed method outperforms state-of-the-art methods on both synthetic and real low-light saturated images and is flexible for both natural and saturated images.

## 2. Related Work

Natural Image Deblurring: The image deblurring methods have been extensively studied as a classical image processing problem [1,2,4,7,9,23,24]. According to whether the blur kernel is known in advance, the image deblurring can be classified into blind image deblurring and non-blind image deblurring. In this work, we focus on non-blind image deblurring. The image prior assumption is the key to non-blind image deblurring. Krishnan et al. [25] employed the hyper-Laplacian $l_{p}$ (0.5≤p≤0.8) to fit the statistical properties of images and solved deconvolution problems with iteratively reweighted least squares (IRLS) for faster speed.

Pan et al. [5] found that blurring destroys the dark channel characteristics of clear images and used dark channel priors to constrain images. Further, Yan et al. [26] observed that bright pixels in clear images would be obviously darkened under blurring effects and proposed extremely bright channel priors for image deblurring. This observation also inspired Chen et al. [27] to propose a local maximum gradient prior. Bai [28] found that large-scale edge gradient distribution had bimodal characteristics, which was destroyed by blurred images, resulting in unimodal distribution.

The weighted total variational priori based on graphs were proposed to promote bimodal gradient distribution of intermediate images. Recently, deep-learning-based methods have also achieved great progress in image deblurring [29,30,31,32,33,34,35].

Tao et al. [31] proposed an end-to-end scale-recurrent network (SRN), which uses image multi-scale information to increase the network receptive field to better capture the global image information. The generative adversarial network (GAN) has been introduced to treat the image deblurring as an image-to-image translation problem with an impressive visual appearance [33]. Most of these methods are derived from the linear blur model, while are not proper for the non-linear degradation of the low-light saturated images.

Low-Light Saturated Image Deblurring: The first category of methods [10,11] implicitly formulates the nonlinear degradation of saturation via detection and deblurring pipelines. They first detected the potential saturation pixels and completely discarded them, which can be regarded as a hard threshold operation to prevent the negative effects on the natural pixels.

In [10,11], the authors detected the saturated pixels of multi-frame images with a predefined threshold and discarded them afterward followed by linear deblurring on the remainder of each frame. However, as the accuracy of detection heavily depends on the threshold, an incorrect threshold can lead to erroneous detection results and, consequently, to the presence of ringing artifacts. In this work, we avoid this cumbersome and error-prone saturation-detection procedure.

The second class of methods [12,13,14,16,17,19,20,21] usually derives a deblurring model based on a modified non-linear blur model that explicitly formulates the saturation truncation. They constructed a deblurring model that assigns the saturated pixels small weights to suppress the errors introduced by saturated pixels. Different from the first category, they explicitly model the saturation in a soft weighted manner.

Specifically, Cho et al. [12] and Zhang [21] regarded the saturated pixels as outliers that violate the linear degradation model and made use of an expectation-maximization (EM) algorithm to iteratively calculate the pixel weights that are proportional to the expectation of the outliers and conducted weighted deconvolution. In [14], Whyte et al. proposed a weighted deconvolution model based on the Richardson–Lucy [36,37] algorithm. Calef et al. [13] proposed an iterative re-weighted maximum likelihood estimation, where the weights are calculated according to the restoration residuals in each iteration.

Similarly, in [16,17], the authors proposed a novel data-fidelity term to suppress the large errors caused by saturated pixels and took advantage of the iterative re-weighted least squares (IRLS) algorithm to handle the saturation. Chen et al. [19] suppressed the affects of saturated pixels by explicitly detecting and eliminating them. Liu et al. [18] introduced surface-aware images prior to eliminating saturated pixels in the intermediate estimation image. Chen [20] directly modeled the saturate clipping as the multiplicative process, and the multiplier was used to weight the importance of different pixels. Overall, these methods assign the saturation small weights that are typically calculated according to the iteration error. To principally model the saturation, we formulated the saturation into a unified nonlinear degradation model.

## 3. The Nonlinear Saturation Deblurring Method

### 3.1. Motivation of the Nonlinear Model for Saturation

In this section, we first analyze the common problems caused by saturation in the process of deblurring. Then, the rationality of the nonlinear model is provided.

#### 3.1.1. Analysis of Ringing Artifacts

One of the major problems in conventional linear deblurring modeling is the presence of ringing artifacts as shown in Figure 1. Ringing artifacts are dark and light ripples that appear near strong edges after deconvolution [1]. It is widely accepted that the ringing artifacts are usually caused by the abrupt step signals in the images, namely the high frequency in the Fourier domain, such as the saturation and the noises [1].

We show the deconvolution results of saturated images to analysis the ringing artifacts as shown in Figure 1. The close-ups in the second row show that the compared deblurring methods produce severe artifacts. Similarly, all of the artifacts are widely distributed around the saturation areas and propagate outwards in a ringing shape. In addition, we present the cross profile along the vertical blue line as shown in the third row of Figure 1. The curves depict the spatial distribution of the intensity.

We observe that the curves of compared methods oscillate, resulting in high-frequency artifacts. The main reason why ringing artifacts are caused is that the compared deblurring methods are based on a linear model, which violates the truth degradation of saturated region. Thus, the linear model introduces restoration errors, and the large errors are spread out from saturated area to the unsaturated area, which looks similar to the ringing artifacts.

To suppress the ringing artifacts, we propose a nonlinear model to accommodate the degradation at saturation pixels. As shown in Figure 1e, fewer ringing artifacts occur in close-ups, and the curve also remains relatively steady and closer to the ground-truth image. The results indicate that an appropriate model to fit the saturation pixels is critical to suppress ringing artifacts.

#### 3.1.2. Estimation Error for Different Regions

**Figure 2:**
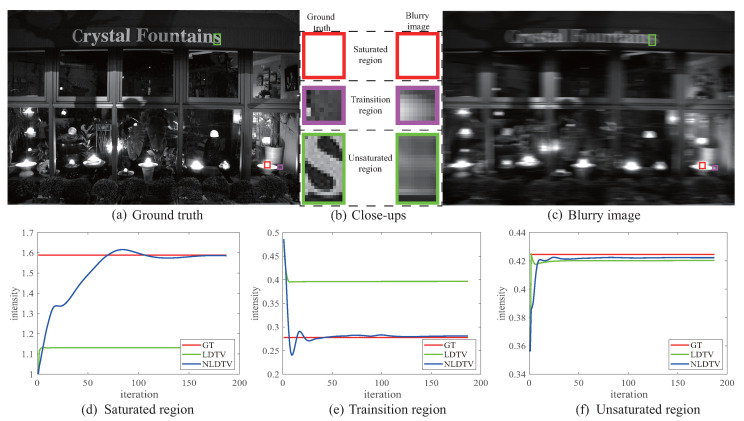
Tracking the average intensity changes of different regions with respect to iteration numbers. Ground-truth and blurry images with saturated, transition, and unsaturated regions marked in boxes are presented in (**a**,**c**). (**b**) is the close-ups of the marked regions, whose first column shows the close-ups of the ground truth and the second ones are the close-ups of the blurry image. The second row presents the average intensity changes of the above three regions (**d**) saturated region, (**e**) transition region, and (**f**) unsaturated region with respect to the iteration numbers. Note that GT means the ground-truth intensity. The nonlinear method approaches the ground truth for different regions while the linear method does not.

We classify a saturated image into three main regions: the saturation, the transition, and the natural regions. We separately illustrate the errors between the estimated value and ground-truth intensity of the three regions as shown in Figure 2. Since no accommodation is adopted for the different regions by the linear deblurring based on total variation (LDTV) [38], we can observe that there exists severe estimation error in the saturation and transition regions (second row in Figure 2).

In detail, for the saturated region, Figure 2d plots the average intensity of the red box, which is marked in Figure 2a,c. With iteration occurring, there exist large errors for LDTV. For the proposed nonlinear model NLDTV, the error reduces to a value close to zero. As for the transition region, Figure 2e plots the average intensity between the saturated area and the unsaturated area (purple box). This exhibits relatively smaller errors for nonlinear deblurring compared with LDTV. The plot of the region in the unsaturated area (green box) is shown in Figure 2f. Both LDTV and our nonlinear model can restore the intensity with high accuracy. Thus, we conclude that the nonlinear model NLDTV can adaptively model three regions with low estimation errors.

#### 3.1.3. Rationality of Nonlinear Model for Saturation

To accommodate different regions with different intensities, we additionally introduce a clipping function on the conventional linear model (1) as follows:

(2) y=c(Kx)+n,

where c(·) is a nonlinear clipping function that shares the same form as in [39]. To analyze the previously mentioned problems, we give an illustration of how the proposed nonlinear model fits the saturation. Note that the noise term is outside the clipping function for simplicity; the influence of noise at saturation regions is slight and can be ignored there.

**Figure 3:**
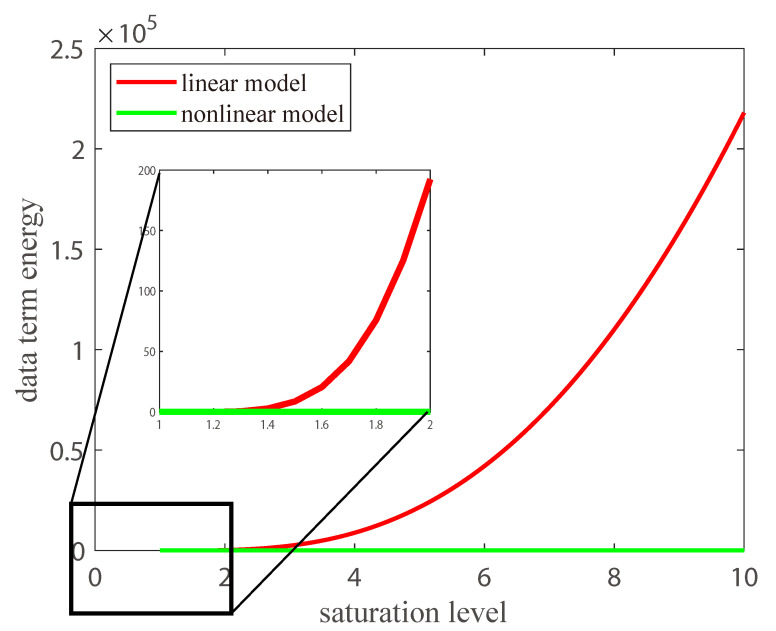
The rationality of our nonlinear model for saturation deblurring. Each curve shows the goodness-of-fit of the data terms at different saturation levels (intensity of saturated pixels). The lower value of energy denotes the greater robustness of the data fidelity for saturation.

Saturation is related to the exposure length, and a long exposure time results in a high saturation level. Thus, we show the energy change of the data-fidelity term with the increase of saturation level as shown in Figure 3. The value of the data-fidelity term of the linear deblurring model increases drastically with the rising saturation level (the red curve). According to the analysis in [40], a good data-fidelity term should remain stable as a low-value, especially for a large error. Thus, the linear model is no longer applicable to saturation. The value of the data-fidelity term of the proposed nonlinear model (the green curve) remains low and nearly constant as the saturation level increases. Such a nonlinear function on the conventional model benefits the capture of the formation process of the saturated image. Therefore, a deblurring method based on the nonlinear model where the data-fidelity term is less sensitive to the saturation would be effective for saturation deblurring.

### 3.2. Nonlinear Deblurring with Total Variation

#### 3.2.1. The Clipping Function

The clipping function is introduced to accommodate the different regions. Thus, the problem of saturation deblurring is transferred into a simpler and specific problem about how to design a reasonable nonlinear function. The intensity range of y can be normalized to between [0, 1]. It is natural for us to define the clipping function as c(x)=min(x,1). This clipping function is quite similar to the rectified linear unit (RELU) function, which is utilized for pursuing sparsity for different regions and endowing the highly nonlinear ability of the neural network.

The motivation of our nonlinear function c(x) is in line with the RELU and is used to differentiate signals in the saturation, transition, and natural regions. For the natural pixels, we keep them the same as in the conventional linear model. For the transition and saturation pixels, we enforce a hard constraint to bound the pixel ranging, thus, prohibiting the error propagation from the saturation to the natural pixels. This is the exact reason why ringing artifacts exist in the conventional linear model.

**Figure 4:**
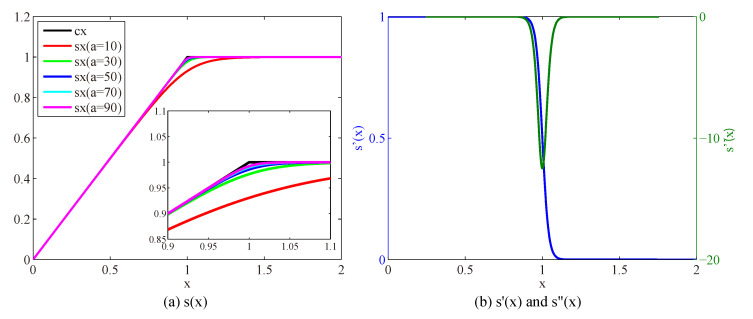
Nonlinear function s(x) and its derivatives. (**a**) Nonlinear function s(x) with different parameter values of *a* and (**b**) the first- and second-order derivatives of s(x) with parameter a=50.

However, the clipping function c(x) is not differentiable at the point of 1, which means the modeling will be intractable for all the saturated pixels. To tackle the non-differentiability of c(x), we replace it with an approximating function s(·) defined as

(3) $$s\left(x\right)=x-\frac{1}{a}\ln\left(1+e^{a(x-1)}\right),$$

where *a* is a scalar that controls the accuracy and the nonlinearity of the model. s(x) is a smooth, continuously differentiable function as shown in Figure 4b.

The accuracy and the nonlinearity increase as parameter *a* becomes larger, as shown in Figure 4a. Higher model accuracy facilitates better restoration performance, while higher model nonlinearity leads to a more difficult optimization process. We set *a* to 50 to achieve a balance between the accuracy and nonlinearity. Thus, an approximating degradation model is formulated as

(4) y=s(Kx)+n.

Note that, the model (4) formulates the degradation process of saturation (reflected in truncated part in Figure 4) and the pixels of unsaturated pixels (reflected in linear part in Figure 4) simultaneously.

#### 3.2.2. The Adaptability of the Approximating Function s(·)

Our nonlinear data item can be deformed into the form of the weighted L2 norm, and the change of weight w is analyzed. According to the property of s(x), as shown in Figure 4a of the main manuscript, when r≤1,s(r)≈r, then we have:

(5) $$w_{i}=\frac{r_{i}-y_{i}}{r_{i}-y_{i}}=1,$$

When r≥1, according to the property of s(x), s(r)≈1,y=1, then we have:

(6) $$w_{i}=\frac{1-y_{i}}{r_{i}-y_{i}}\to 0,$$

That is, in the deconvolution process, we hope that, for unsaturated pixels, the weight is 1; for saturated pixels, the weight is close to 0, thus, avoiding the ringing diffusion caused by the inaccurate estimation of saturated pixels. Through the following experiments, we found that, as the iteration progresses, the value of weight w gradually conforms to the above analysis, indicating that our algorithm gradually accurately allocates weights, thus, achieving the effect of adaptively modeling saturated and unsaturated areas.

**Figure 5:**
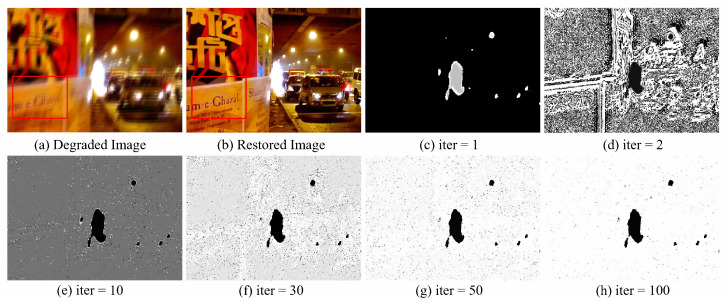
(**a**) The degraded image, (**b**) the restored image, and (**c**–**h**) variations of the weight W with iterations.

It can be observed in Figure 5 that, as the iterations progress, the weight W gradually approaches 1 for non-saturated region pixels and approaches 0 for saturated pixels, indicating a gradual improvement in modeling different region pixels accurately. Moreover, in the final iteration results, the values of W correspond more accurately to the saturated/non-saturated pixels in the image, which indicates that our proposed non-linear deblurring method can adaptively process different pixels in the image.

#### 3.2.3. The Proposed NLDTV Model

Based on Equation (4) and maximum a posteriori framework, the proposed nonlinear deconvolution framework can be formulated as

(7) $$\hat{x}=\arg\min_{x}\frac{1}{2}||s\left(\mathrm{Kx}\right)-{y||}_{2}^{2}+\lambda \phi \left(x\right).$$

Different image priors used in the framework will lead to different deconvolution methods. Making use of the isotropic total variation [41], we propose the nonlinear deblurring based on total variation (NLDTV), which is to solve the following optimization problem

(8) $$\hat{x}=\arg\min_{x}\frac{1}{2}||s\left(\mathrm{Kx}\right)-{y||}_{2}^{2}+\lambda \sum_{p}{||\left(\mathrm{Dx}\right)}_{p}{\left|\right|}_{2},$$

where *p* is the pixel index, $D:=[D_{h};D_{v}]$ is a combination of the first-order finite difference operators $D_{h}$ and $D_{v}$ with respect to the horizontal direction and the vertical direction, and ${\left(\mathrm{Dx}\right)}_{p}=\left[{\left(D_{h}x\right)}_{p};{\left(D_{v}x\right)}_{p}\right]$ is the gradient vector of the $p^{th}$ pixel in x, in which ${\left(D_{h}x\right)}_{p}$ and ${\left(D_{v}x\right)}_{p}$ are the horizontal and vertical gradients, respectively.

### 3.3. Numerical Optimization

The proposed deblurring method exploits a component-wise nonlinearity s(x) to approach the clipping function c(x). Although the s(x) is a smooth and continuously differentiable function, the nonlinearity also hinders us to solve the problem. To deal with the nonlinearity, we combine with optimization strategy of [39], and optimize the proposed deblurring method with ADMM.

Let d=Dx and r=Kx; the optimization problem (8) can be reformulated as

(9) $$\begin{array}{cc} \; & \left\{\hat{d},\hat{r},\hat{x}\right\}=\arg\min_{d,r,x}\frac{1}{2}||s\left(r\right)-{y||}_{2}^{2}+\lambda \sum_{p}\left|\right|d_{p}{\left|\right|}_{2}\\ \; & +\frac{\rho_{r}}{2}||\mathrm{Kx}-r+\frac{u_{r}}{\rho_{r}}{\left|\right|}_{2}^{2}+\frac{\rho_{d}}{2}||\mathrm{Dx}-d+\frac{u_{d}}{\rho_{d}}{\left|\right|}_{2}^{2}, \end{array}$$

where $\rho_{d}$ and $\rho_{v}$ are the weights of the punishing terms and $d_{p}=\left[{\left(d_{h}\right)}_{p};{\left(d_{v}\right)}_{p}\right]$, and $u_{r},u_{d}$ are the Lagrangian multipliers to the constrains. Fixing the independent variables, the optimization problem (9) can be divided into the following subproblems of d, r, and x

(10) $$\begin{array}{cc} \hat{d} & =\arg\min_{d}\lambda \sum_{p}\left|\right|d_{p}{\left|\right|}_{2}+\frac{\rho_{d}}{2}||\mathrm{Dx}-d+\frac{u_{d}}{\rho_{d}}{\left|\right|}_{2}^{2}, \end{array}$$

(11) $$\begin{array}{cc} \hat{r} & =\arg\min_{r}\frac{1}{2}||s\left(r\right)-{y||}_{2}^{2}+\frac{\rho_{r}}{2}||\mathrm{Kx}-r+\frac{u_{r}}{\rho_{r}}{\left|\right|}_{2}^{2}, \end{array}$$

(12) $$\begin{array}{cc} \hat{x} & =\arg\min_{x}\frac{\rho_{r}}{2}||\mathrm{Kx}-r+\frac{u_{r}}{\rho_{r}}{\left|\right|}_{2}^{2}+\frac{\rho_{d}}{2}||\mathrm{Dx}-d+\frac{u_{d}}{\rho_{d}}{\left|\right|}_{2}^{2}. \end{array}$$

In the following optimization process of subproblems, the superscripts *i* and *j* indicate the iteration times of different loops. The subproblem (10) of d can be solved using the generalized soft threshold operator

(13) $$\left({\left(d_{\mathrm{h-italic}}\right)}_{p}^{i+1},{\left(d_{v}\right)}_{p}^{i+1}\right)=S_{\frac{\lambda }{\rho_{d}}}\left({\left(D_{h}x\right)}_{p},{\left(D_{v}x\right)}_{p}\right),$$

where $\left(y_{1},y_{2}\right)=S_{t}\left(x_{1},x_{2}\right)$ is defined as

(14) $$y_{q}=\frac{x_{q}}{\sqrt{x_{1}^{2}+x_{2}^{2}}}\cdot \max\left(\sqrt{x_{1}^{2}+x_{2}^{2}}-t,0\right),q\in \left\{1,2\right\}.$$

There is not a closed-form solution to the subproblem (11) of r; however, it can be iteratively solved with the Newton Method, due to the nonlinear function s(·) being continuously differentiable. The iterating equation is expressed as

(15) $$r^{j+1}=r^{j}-\frac{L^{\prime }\left(r^{j}\right)}{L^{\prime \prime }\left(r^{j}\right)},$$

where $L^{\prime }\left(r\right)$ and $L^{\prime \prime }\left(r\right)$, defined below, are the first-order derivate and the second-order derivate of the energy function (11), respectively,

(16) $$\begin{array}{cc} L^{\prime }\left(r\right) & =s^{\prime }\left(r\right)\left(s\left(r\right)-y\right)+\rho_{r}\left(r-{\mathrm{Kx}}^{i}\right)-u_{r}^{i}, \end{array}$$

(17) $$\begin{array}{cc} L^{\prime \prime }\left(r\right) & =s^{\prime \prime }\left(r\right)\left(s\left(r\right)-y\right)+s^{\prime }{\left(r\right)}^{2}+\rho_{r}. \end{array}$$

The process of solving (11) with the Newton Method is summarized in Algorithm 1.
**Algorithm 1** Solve Subproblem of r with the Newton Method**Require:** 
$r^{i},y,\lambda$1:j←0, ε←1, $r^{j}\leftarrow r^{i}$2:**while**$\varepsilon >\tau_{r}$ and $j<\upsilon_{r}$ **do**3:   calculate $L^{\prime }\left(r^{j}\right)$ and $L^{\prime \prime }\left(r^{j}\right)$ via Equations (16) and (17);4:   update $r^{j+1}$ with Equation (15);5:   calculate $\varepsilon^{j}$ using $\frac{\left|\right|r^{j+1}-r^{j}\left|\right|}{\left|\right|r^{j+1}\left|\right|}$;6:   j←j+1.7:**end while**8:**return **$r^{i+1}$.

The subproblem (12) of x is of quadratic form and can be solved by solving the following linear system equation,

(18) $$\left(\rho_{d}D^{⊤}D+\rho_{r}K^{⊤}K\right)x=D^{⊤}\left(\rho_{d}d-u_{d}\right)+K^{⊤}\left(\rho_{r}r-u_{r}\right),$$

and closed-form solution as follows can be obtained with fast Fourier transform (FFT), assuming circular boundary conditions,

(19) $$x^{i+1}=F^{-1}\left(\frac{F(D^{⊤}\left(\rho_{d}d^{i+1}-u_{d}^{i}\right)+K^{⊤}\left(\rho_{r}r^{i+1}-u_{r}^{i}\right))}{F(\rho_{d}D^{⊤}D+\rho_{r}K^{⊤}K)}\right),$$

where F(·) and $F^{-1}\left(\cdot \right)$ are Fourier transform and inverse Fourier transform, respectively. Finally, the Lagrangian multipliers are updated as follows,

(20) $$\begin{array}{c} u_{d}^{i+1}=u_{d}^{i}+\rho_{d}\left({\mathrm{Dx}}^{i+1}-d^{i+1}\right), \end{array}$$

(21) $$\begin{array}{c} u_{r}^{i+1}=u_{r}^{i}+\rho_{r}\left({\mathrm{Kx}}^{i+1}-r^{i+1}\right). \end{array}$$

The algorithm scheme of NLDTV is summarized in Algorithm 2.
**Algorithm 2** Nonlinear deblurring based on total variation (NLDTV)**Require:** 
y,k,λ1:i←0, ε←1, $f^{i}\leftarrow y$, $r^{i}\leftarrow {\mathrm{Kx}}^{i}$, $u_{d}^{i}\leftarrow 0$, $u_{r}^{i}\leftarrow 0$;2:**while**ε>τ and i<υ **do**3:   calculate $d^{i+1}$ with Equation (13);4:   solve for $r^{i+1}$ via Algorithm  1;5:   calculate $x^{i+1}$ using Equation (19);6:   update $u_{d}^{i+1}$ and $u_{r}^{i+1}$ via Equations (20) and (21);7:   calculate ε using $\frac{\left|\right|x^{i+1}-x^{i}\left|\right|}{\left|\right|x^{i+1}\left|\right|}$;8:   i←i+1.9:**end while**10:**return **x.

## 4. Experimental Results

### 4.1. Experimental Setting

To evaluate the effectiveness of the proposed method, we examine our algorithm on both synthetic [42] and real-world image datasets [12] with significant saturation outliers. The synthetic dataset is created by ten ground-truth low-light images (The images are collected from website www.flickr.com/photos/oimax (accessed on 1 May 2022)) and ten blur kernels. The ten blur kernels include eight motion blur kernels provided by [4]. Specifically, each ground-truth image is extended to a high dynamic range scaled by a saturation level factor, τ, and then is synthetically blurred by ten kernels.

We mask the high intensity pixel with the threshold t=0.9 and multiply with the saturation level factor τ. Note that, the high-intensity pixels of each blurry image are clipped by clipping function c(∗), and the intensity range of the image is normalized to the range of [0, 1]. Moreover, we also add noise level σ= 0.01 random noise on each blurry image. We compare the proposed method with four deconvolution methods, including:

- SOTA linear deblurring methods RGTV [28] and DCP [5].
- SOTA model-based low-light saturated image deblurring methods of Whyte [14], Cho [12], Chen [19].
- SOTA learning-based low-light saturated image deblurring methods NBDN [35].

For fair comparisons, we use the original implementations of the compared methods, set the iteration numbers of RL to 50, and retrain the learning-based method NBDN [35] on our synthetic dataset. In our experiments, the tolerance τ and threshold of iteration times υ in Algorithm (2), and, $\tau_{r}$ and $\upsilon_{r}$ in Algorithm (1) are set to be $10^{-4}$ and 1000 and $10^{-4}$ and 2.

### 4.2. Non-Blind Deblurring Results

#### 4.2.1. Restoration Results on Simulated Datasets

To quantitatively evaluate the performance of the proposed method, we test the algorithms on synthetic blurry images. The tested images are generated from dataset Exdark [42]. Table 1 lists the reference-based indexes of PSNR (: dB) and SSIM and non-reference-based index BRISQUE of different methods for the images blurred with motion kernels. As we can see, the proposed method achieves favorable results against the compared methods in terms of PSNR and SSIM values. In addition, we present the visual performance of different methods on the synthetic datasets of motion blur.

**Figure 6:**
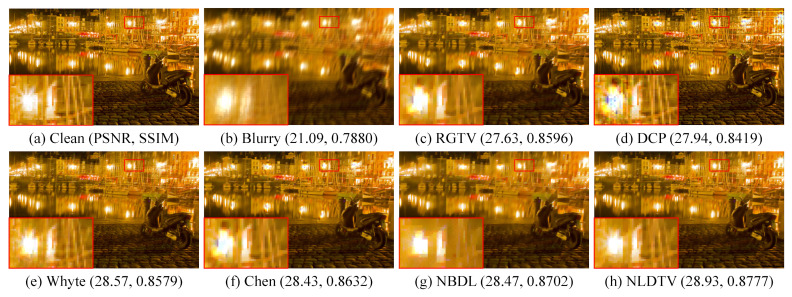
Qualitative evaluation on saturation dataset with motion blur. (**a**) Clean image, (**b**) blurry image, and (**c**–**h**) restored by RGTV [28], DCP [5], Whyte [14], Chen [19], NBDN [35], and the proposed NLDTV.

Figure 6c,d shows that the linear deblurring methods, including RGTV [28] and DCP [5], cause serve artifacts around saturation areas. The low-light saturated image deblurring methods, including those of Whyte [14], Cho [12], and Chen [19], can handle images with saturated areas. However, Whyte [14] is less effective to recover the salient structures as shown in Figure 6e. Cho [12] and Chen [19] tend to produce color artifacts at saturated regions as shown in Figure 6f,g.

On the contrary, the proposed method can deal with saturated areas and effectively suppresses the ringing artifacts. Compared with existing saturation deblurring methods, NLDTV obtains favorable visual performance for removing blur and preserving details as shown in Figure 6h. Overall, the proposed method outperforms the state-of-the-art algorithms in terms of quantitative and qualitative evaluation of the synthetic datasets.

#### 4.2.2. Robustness to Different Saturation Levels

**Figure 7:**
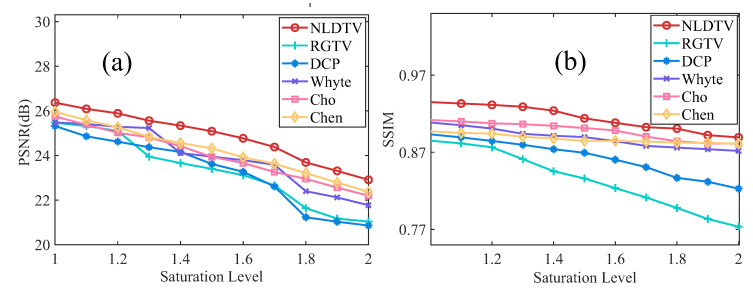
Effectiveness evaluation of different saturation levels. With different saturation levels τ=[1.0,2.0], we show the (**a**) PSNR and (**b**) SSIM values of different methods.

To evaluate the influence of saturation level, we present the results of different methods with saturation levels τ = [1.0, 2.0] as shown in Figure 7. As we can see, the performances of all methods decrease with the increasing of saturation level; however, the proposed method can consistently obtain the best results in each saturation level. Moreover, the decreasing tendency of each method represents the robustness of the saturation level. The NLDTV obtains the lower decreasing ratio, which means less influence with the increasing of saturation level, demonstrating that NLDTV is the general method for saturation and is robust to different saturation levels.

### 4.3. Blind Deblurring Results

#### 4.3.1. Combining with Kernel Estimation Method

**Figure 8:**
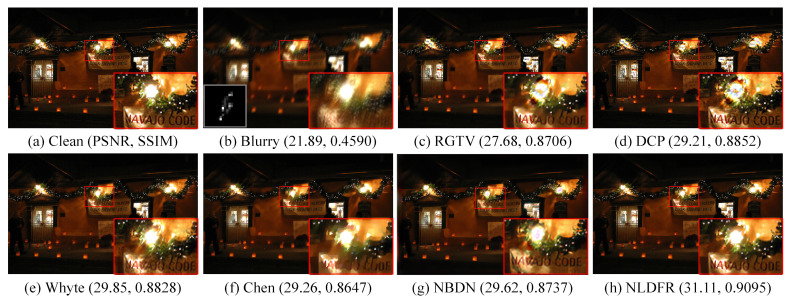
Results of blind deblurring with the estimated kernel. (**a**) Clean image, (**b**) blurry image, and (**c**–**h**) restored by RGTV [28], DCP [5], Whyte [14], Chen [19], NBDN [35], and the proposed NLDTV.

We recover images with the kernel, which is estimated by other kernel estimation methods, to further evaluate the flexibility of the proposed method. Figure 8 shows the results of non-blind deblurring based on the kernel estimated by [17]. The NLDTV achieves the best performance in terms of PSNR and SSIM. The linear methods, including RGTV [28] and DCP [5] produce serve ringing artifacts in saturation, while Cho produces artifacts in the natural region due to the inaccurate kernel. The saturation deblurring methods Cho, Whyte, Chen, NBDN, and NLDFR obtain high-quality restored results.

Note that the learning-based method NBDN can restore the details effectively but may generate color artifacts in the saturated regions. NLDTV restores the finer details compared to other saturation deblurring methods, especially for the texts in the red close-ups. The result indicates that the proposed method can restore the images by combining with the existing kernel estimation methods.

#### 4.3.2. Deblurring Real-World Image

**Figure 9:**
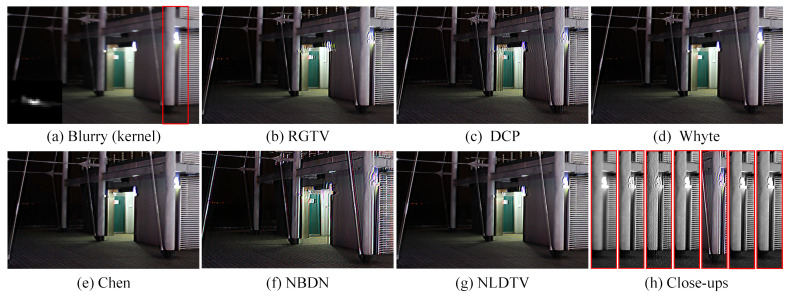
Results of deblurring a real-world saturated image. (**a**) Blurry image and estimated blur kernel and (**b**–**g**) restored by RGTV [28], DCP [5], Whyte [14], Chen [19], NBDN [35], and the proposed NLDTV. (**h**) Close-ups.

Furthermore, we ran real-world experiments to test the effectiveness of the proposed method. The blurry image and estimated kernel are collected from [12]. As shown in Figure 9, there are just a few ringing artifacts in the restored image of the saturation-based methods, while there are significant artifacts in the restored images of RGTV [28] and DCP [5]. For the convenience of comparison, the saturation areas cropped from results are displayed in Figure 9h successively. The proposed methods can perfectly suppress the ringing artifacts and restore the blurry image with pleasant visual quality in the real case.

### 4.4. Discussion

#### 4.4.1. Effectiveness for the Low-Light Saturated Image Object Detection

**Figure 10:**
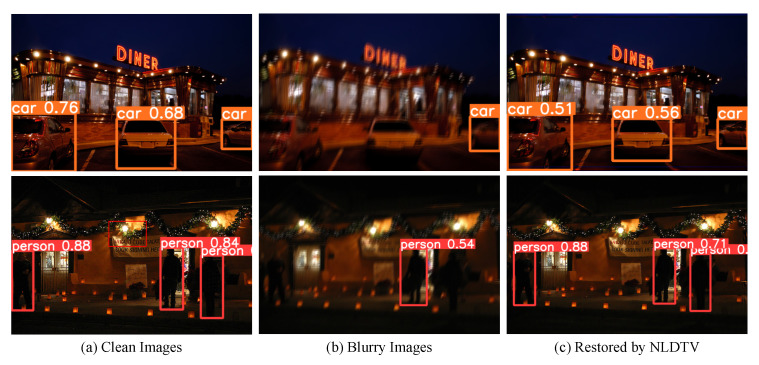
Object detection results of clean images, blurry images, and restored images by the Yolov7 [43]. The confidences of the detected objects, from left to right, are listed in Table 2.

We used the SOTA object-detection method Yolov7 [43] to demonstrate that the restored image by NLDTV can facilitate the object detection performance. We compared the detection performance on the clean image, degraded image, and restored image by the pre-trained Yolov7 model, and the results are shown in Figure 10. The yolov7 can detect the can and person object with high confidence as shown in Figure 10a. However, the detection performance dramatically dropped at the blurry image (Figure 10b). For the car object, an apparent white car was missed by the Yolov7, and two-person objects were also missed.

The detected person object has only 54% confidence. These state that the blur and saturation degradation may heavily distort the objects or disturb the detection model. After deblurring by the proposed NLDTV, the accuracy of detection gained a large increase as shown in Figure 10c. The missing cars and persons awere detected by the Yolov7, and the confidence also increased. The detection comparison illustrates that the proposed NLDTV can boost the high-level object detection task on low-light and blurry images.

#### 4.4.2. The Impact of the Parameters

**Figure 11:**
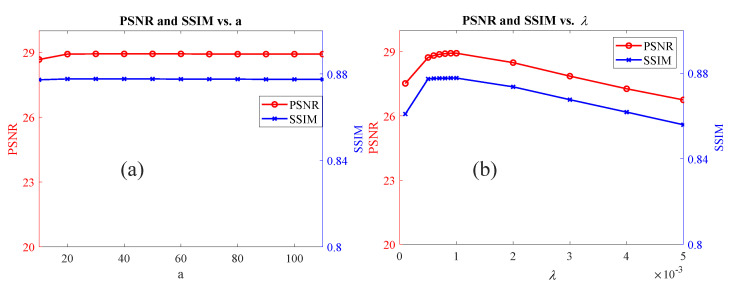
Variation of PSNR and SSIM values with changes in the parameters a (**a**) and λ (**b**) for image restoration.

- **The impact of `a’**: We performed a sensitivity analysis to investigate the impact of ’a’ on the overall performance of our proposed method, the results are shown in Figure 11a. The line chart of Figure 11a illustrates the variations of PSNR and SSIM values with respect to the parameter ’a’. It is evident from the chart that both PSNR and SSIM are quite robust to changes in the ’a’ parameter. Despite some minor fluctuations in the PSNR and SSIM values, they remain relatively constant throughout the range of ’a’ values. This suggests that the choice of ’a’ has little effect on the performance, and the algorithm is able to produce consistent results across a range of ’a’ values.
- **The robustness to λ**: We analyzed the impact of the key regularization parameter λ on the performance of the proposed algorithm and evaluated the robustness of the algorithm to λ. The results are shown in the line chart of Figure 11b. From the line chart, it can be observed that there is a certain degree of variation in both PSNR and SSIM values with changes in the lambda parameter; however, the magnitude of these changes is relatively small, indicating that both metrics are quite robust to the lambda parameter. Particularly in the interval where lambda values are greater than 0.001 and less than 0.01, the changes in PSNR and SSIM are relatively small, indicating that lambda has a stable impact on image restoration performance within this range.

#### 4.4.3. Convergence

**Figure 12:**
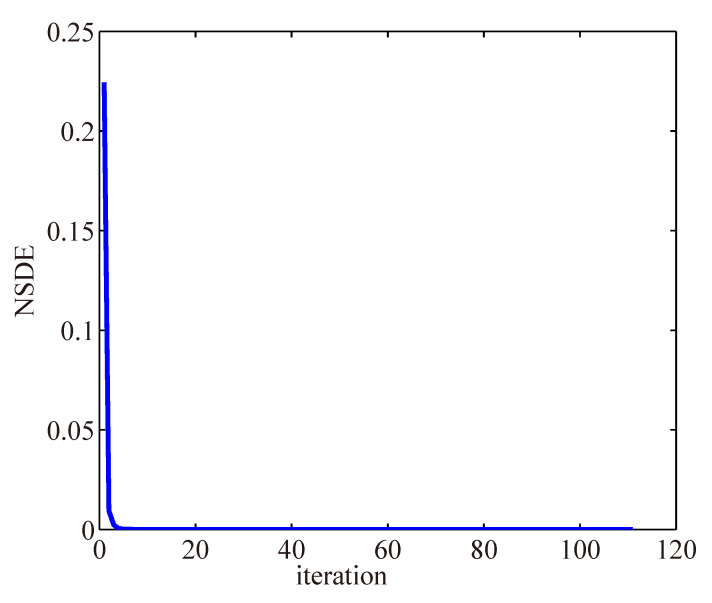
Convergence property of proposed method. The horizontal axis and vertical axis represent the iteration numbers and NSDE, respectively.

To validate the convergence of the proposed method, we studied the normalized step difference energy (NSDE), defined as $\left|\right|f^{k+1}-f^{k}{\left|\right|}_{2}^{2}/\left|\right|f^{k+1}{\left|\right|}_{2}^{2}$, of the proposed method. The NSDE curve is plotted in Figure 12. The NSDE decreases significantly at the first several iterations and converges after about 100 iterations. It denotes that the proposed method can converge to the constant with fast speed.

#### 4.4.4. Effectiveness for Unsaturated Natural Image

**Figure 13:**
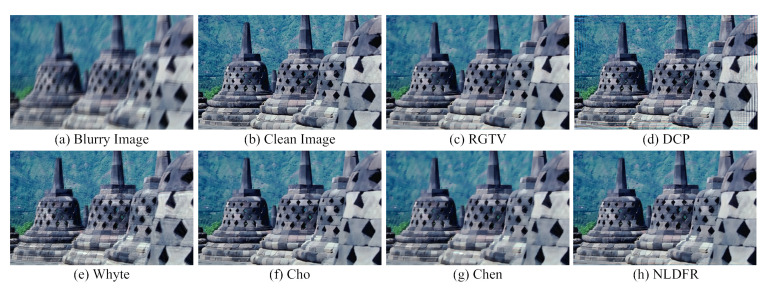
Results of deblurring natural image. (**a**) Clean image, (**b**) blurry image, and (**c**–**h**) restored by RGTV [28], DCP [5], Whyte [14], Cho [12], Chen [19], and the proposed NLDTV.

Although the proposed method focuses on deblurring low-light saturated image, it is also suitable for unsaturated natural image deblurring, due to the unified formulation of saturated pixels and unsaturated pixels. As shown in Figure 13, we validate the effectiveness on natural image suffered from heavy blurry. The proposed method NLDTV achieves the same high quality as representative linear methods RGTV [28] and DCP [5]. Therefore, the proposed method is also effective for both natural and saturated image.

#### 4.4.5. Limitations

The proposed method focused on non-blind image deblurring, and the kernel was given or estimated by other algorithms [12,17]. The accuracy of kernel unavoidably influences the performance of the proposed method. Although this may seem to be limited, the majority of blind deblurring algorithms have a non-blind subcomponent, alternating between kernel estimation and non-blind deblurring. Therefore, the proposed nonlinear formulation can also be introduced to existing blind deblurring framework [7,23,44] to obtain a more accurate kernel.

In addition, although the proposed method achieved state-of-the-art performance, it is limited to uniform blur. In the practice, camera shake and dynamic scene always result in non-uniform blur. Thus, our future works will focus on blind saturated image deblurring and then extend to non-uniform deblurring. Moreover, the saturated images are often shot at low light environments, where would also suffer from random noise. Thus, saturation image deblurring in the presence of the strong noise is also will be studied in the future.

## 5. Conclusions

In this paper, we studied the problem of deblurring saturated images. We observed that there were severe ringing artifacts in the restored image of linear deblurring methods. To solve the problem, we analyzed the nonlinear deblurring model, which outperformed the linear deblurring model regarding the robustness of data-fidelity, reducing the estimation error and suppressing ringing artifacts for saturation deblurring. Furthermore, we proposed a nonlinear degradation model, which included the process of deblurring images with saturated and unsaturated pixels in a unified manner.

Based on the nonlinear degradation model, we proposed a nonlinear deblurring model that can be solved with the ADMM. Our extensive experimental results demonstrate the proposed method outperformed the state-of-the-art methods on both synthetic and real-world images. Moreover, the proposed NDLTV can promote high-level object detection tasks on saturated blurry images in low-light environments.

## Figures and Tables

**Table 1:** Quantitative comparison on synthetic dataset Exdark [42] with motion blur. The reference-based indexes of PSNR (: dB) and SSIM and non-reference-based index BRISQUE of different images are presented, and the last row presents the average (Ave.) indexes of restored results of different methods.

Index	Blurred	RGTV [28]	DCP [5]	Whyte [14]	Chen [19]	NBDN [35]	NLDTV
PSNR ↑	19.13	20.23	20.09	21.30	24.24	23.52	**24.46**
SSIM ↑	0.6668	0.7287	0.7401	0.7486	0.8048	0.7777	**0.8124**
BRISQUE ↓	48.88	39.94	43.85	39.43	25.70	27.25	**24.09**

**Table 2:** The confidences of the detected objects (from left to right) in Figure 10.

Objects	Clean	Blurry	NLDTV
Car	(0.76, 0.68, 0.62)	(0, 0, 0.41)	(0.76, 0.68, 0.48)
Person	(0.88, 0.84, 0.78)	(0, 0.54, 0)	(0.76, 0.68, 0.54)

## Data Availability

Not applicable.
